# Acid and Alkaline Phosphatase Levels in GCF during Orthodontic Tooth Movement

**Published:** 2015-09

**Authors:** Mohammad Farahani, Seyed Mohammadreza Safavi, Omid Dianat, Somayeh Khoramian Tusi, Farnaz Younessian

**Affiliations:** a Dept. of Orthodontic, Dental School, Shahid Beheshti University of Medical Sciences, Tehran, Iran.; b Dental Research Center, Research Institute of Dental Sciences, Shahid Beheshti University of Medical Sciences, Tehran, Iran.; c Dept. of Endodontic, Dental School, Shahid Beheshti University of Medical Sciences, Tehran, Iran.; d Dept. of Pediatric, Dental School, Alborz University of Medical Sciences, Karaj, Iran.; e Dentofacial Deformities Research Center, Research Institute of Dental Sciences, Shahid Beheshti University of Medical Sciences, Tehran, Iran.

**Keywords:** Gingival crevicular fluid, Alkaline phosphatase, Acid phosphatase, Bone deposition, Bone resorption

## Abstract

**Statement of the Problem:**

The present constituents of gingival crevicular fluid (GCF) can reflect the changes occurring in underlying tissues. Considering variety of biologic bone markers, alkaline phosphatase and acid phosphatase have been examined as bone turn over markers in orthodontic tooth movement.

**Purpose:**

The current study designed in a longitudinal pattern to determine the changes of acid and alkaline phosphatase (ACP & ALP) in GCF during orthodontic tooth movement.

**Materials and Method:**

An upper canines from twelve patients (mean age: 14±2 years) undergoing extraction orthodontic treatment for distal movement served as the test tooth (DC), and its contralateral (CC) and antagonist (AC) canines were used as controls. The CC was included in orthodontic appliance without orthodontic force; the AC was free from any orthodontic appliance. The GCF around the experimental teeth was harvested from mesial and distal tooth sites immediately before appliance placement (T0), and 14 (T2) and 28 days (T3) after it and ALP and ACP concentration were determined spectrophotometrically.

**Results:**

ALP concentration was elevated significantly in DC and CC groups at days 14 and 28 compared with the AC. In DC group, the ALP was significantly greater in mesial sites than distal site, while no significant changes were found between both sites of CC. The peak level of ALP was observed in mesial sites of DC at T2. Regarding ACP, significant elevation of this enzyme was seen in DC group both in mesial and distal sites at T2 and T3. The peak level of this enzyme was seen at T2.

**Conclusion:**

Monitoring simultaneous changes of ALP and ACP levels in GCF can reflect the tissue responses occur in periodontium during bone formation and bone resorption during orthodontic tooth movement, respectively.

## Introduction

By increasing the number of patients seeking orthodontic treatment and correction of dentofacial deformities, investigations on biologic basis of orthodontic movement seem absolutely necessary.(1) Several investigations have evaluated the molecular basis of soft and hard tissue responses following orthodontic tooth movement.(2) By precise evaluation of the biologic response of orthodontic forces, the orthodontic force application could be based on each individual tissue responses and therefore the final effect of orthodontic treatment might be improved.(3) In addition, the main problem of retention can be solved to some extent by considering bone turnover rates around each experimental tooth.(3) However, providing a simple non-invasive method is required for achieving these possibilities. In recent years, a few constitutes of gingival crevicular fluid (GCF) have been shown to be diagnostic biomarkers of active tissue destruction in periodontal disease which might also serve as diagnostic markers of biologic responses in orthodontic tooth movement.(4-5)

GCF is an osmotically mediated inflammatory exudate and its constituents are extracted from different sources including microbial dental plaque, inflammatory cells, host tissue, and particularly serum.(5) Many investigations have demonstrated that GCF constituents can be useful for determination of local biological and biomechanical processes associated with metabolic bone turnover during orthodontic tooth movement.(2, 6-10) Different assumptions have been suggested to explain the biological basis of orthodontic tooth movement. Recently, the studies suggest that the mechanical stimulants like orthodontic tooth movement may induce some subsequent inflammatory responses in periodontal tissue.(5, 11-12) The cells can release enough amounts of chemical mediators into GCF; therefore, the amount of these substances may increase during orthodontic tooth movement in GCF.(13) Considering variety of biologic bone markers, alkaline phosphatase (ALP) and acid phosphatase (ACP) have been examined as bone turn over markers in orthodontic tooth movement.(4-5,12, 14-15)

ALP is a glycoprotein thought to be involved in processes leading to mineral formation in tissue like bone and cementum.(15) Robinson (1923) was first in suggesting that the ALP is important in the mineralization of bone and calcifying cartilages.(16) The enzyme is thought to release phosphate ions from organic phosphate esters as a result of which supersaturation would occur, leading to the precipitation of calcium phosphate salts.(15) This enzyme is produced mainly by neutrophils at GCF, although it is also produced by variety of cells including fibroblasts, osteoblasts, and osteoclasts.(6)

ACP is also an enzyme thought to be involved in processes of mineral deformation in tissues like bone. Resorbing cells, such as osteoclasts and macrophages, have been shown to have high ACP activities.(14, 17)

Little information is available in the field of production of these chemical mediators during orthodontic tooth movement. For the first time, Keeling *et al.* (1993) examined tartrate-resistance acid phosphatase (TRAP) changes in serum and alveolar bone during orthodontic tooth movement in 288 adult male rats.(14) The authors reported that enzyme activities increased during orthodontic tooth movement. In addition, high levels of ACP were detected on first day in serum and on third day in bone, whereas the peak of ALP activity in bone has occurred on seventh day. Insoft *et al.* (1996) also described the activity of both phosphatase on longitudinal study with three cases (as split mouth design) and on a cross-sectional design on 30 patients undergoing orthodontic treatment.(4) An increase in GCF ALP activity during orthodontic tooth movement occurred from 1 to 3 weeks of treatment and the peak of ACP activity occurred from 3 to 6 weeks. It is worth mentioning that this studying was the only investigations that have evaluated the ACP activity changes during orthodontic tooth movement in human subjects.(4) However, the design of the study was based on cross-sectional study without controls which is considered as the distinct methodological shortcoming; therefore, the achieved data could not be interpreted without cautions.(5)

Parinetti *et al.* (2002) have examined ALP activity in gingival crevicular fluid during orthodontic tooth movement and demonstrated that the ALP activity was significantly higher in tension sites rather than compression sites of teeth undergoing orthodontic forces.(5) Moreover, a positive correlations between GCF ALP activity and subgingival colonization of *action bacillus actinomycetemcomitan *s (Aa) has been reported.(18)

According to the restricted number of studies that have investigated the changes in levels of ALP and ACP activities during orthodontic tooth movement, the current longitudinal study was aimed to evaluate the both ALP and ACP activities changes in gingival crevicular fluid during orthodontic tooth movement. 

## Materials and Method

Twelve orthodontic patients (7 female and 5 male individuals; age range 11-17 years) were participated in this study. The protocol of the study was reviewed and approved by the Ethical Committee of Shahid Beheshti University of Medical Sciences. The below inclusion criteria were considered:

1. The requirement of first-premolar extraction and canine distal tooth movement as a part of fixed orthodontic treatment regimen.
2. Healthy periodontium with general probing depth of < 3mm.
3. A full-mouth plaque score (FMPS) of <20% that was measured by dividing the supra gingival plaque score from total tooth surfaces and multiplied by 100.
4. A full mouth bleeding score (FMBS) <20% that was measured by dividing the bleeding after probing from total surfaces multiplied by 100.

The exclusion criteria were deliberated as:

1. History of systemic or infective disease or antibiotic therapy within the past 1 month and not pregnant.
2. History of consumption of anti-inflammatory drugs during the month preceding the study.
3. Radiographic evidence of periodontal bone loss.
4. Probe depth >3mm which was measured with 20-g controlled force by Jensen JP-1 probe (Jensen Dental manufacturer) in 6 sites per tooth.
5. Disagreement in participating in the study presented by an informed consent.

All subjects received oral hygiene instructions (OHIs) for the correct usage of tooth brush and dental floss and were given two bottle of mouthwashes containing chlorhexidine (Shahr Darou Co.; Tehran, Iran) for using twice per day to minimize the possible role of gingival inflammation on changing enzymatic activities. Before the beginning of the study, informed consents were obtained from all participants or guardians (for patients under 16 years of age). 

**Experimental and Control groups**

**Figure 1:**
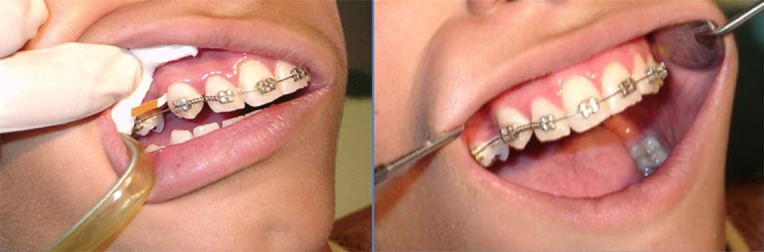
Orthodontic appliance placement setting and GCF collection of one sample.

Twelve patients (mean age: 14±2 years) after passing at least 7-10 days from the time of the extraction of the maxillary first premolar were participated in this study. In each patient, maxillary canines underwent orthodontic treatment. One of the upper canines, the distalized canine (DC) was to be treated for distal movement and was used as the experimental tooth. The contralateral canine (CC) was received the orthodontic appliances but was not moved distally and thus used as a positive control tooth. The antagonist canine (AC) was not received any orthodontic appliance considered as a negative control group. The DC and CC groups were assigned randomly by using toss of coin technique in each patient (Figure 1).

**Orthodontic treatment**

**Figure 2:**
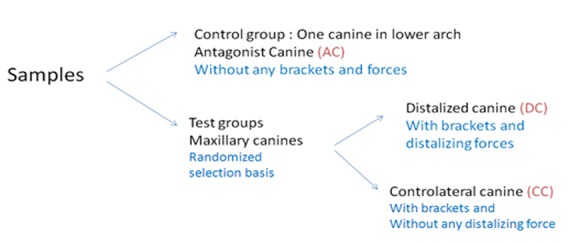
Schematic demonstration of sample classification of experimental and control groups.

Orthodontic pre-adjusted brackets (Roth; 3M Unitek, Monrovia, CA) were bonded on the buccal surfaces of teeth of DC and CC groups. In both arches, the first molars were banded. A 0.014-inch circular nickel-titanium wire (3M UNITEK; Monrovia, CA) was used to initiate the orthodontic movement. A light NiTi open coil spring (American Orthodontics; Sheboygan, WI USA) exerting a constant force (approximately 250 g; measured by force gauge) over its range of activation was included in the appliance between maxillary lateral incisors and canines to retract the experimental canines at DC groups. The length of the coil spring was set as one width bracket more than the space between two adjacent brackets of laterals and canines (Figure 2).

**GCF collection and clinical indices monitoring**

GCF was collected from the mesial and distal aspects of the DC, CC and AC teeth for the ALP and ACP activity assay. These procedures were done immediately before the appliance placement and activation (T1), and at 2 (T2) and 3 (T3) weeks after placement. For determination of interfering variables, the plaque index (PL) was recorded before the GCF collection and then probing depth (PB) and the presence and absence of bleeding on probing (BoP) were recorded after GCF collection. 

**Figure 3:**
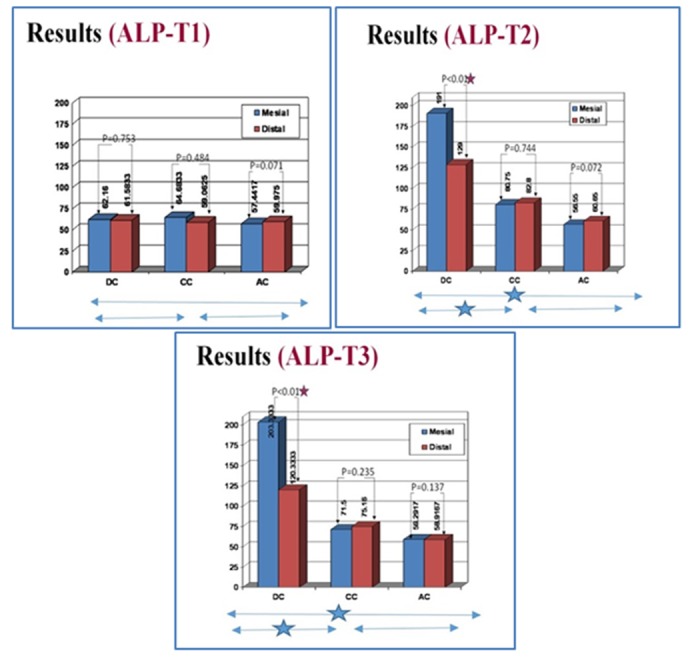
ALP activity (mean) in GCF at mesial and distal sites of DC, CC and AC groups (n=12) at three time points (T1,T2 and T3).

The teeth were carefully cleaned with cotton pellets and a gentle air stream was directed toward the tooth surface for 15 seconds to dry the area. Consequently, single sample of GCF was collected from the selected areas by means of paper strips (Periopaper; Proflow Amityville, NY) inserted 1mm into the gingival crevice and left for 30 seconds (Figure 3). This method of GCF collection has proven to benefit from several advantages over other previous methods including being efficient and less disturbing.(19-20) The operator tried to avoid any mechanical injury. Paper points were transferred to labeled plastic vials immediately after collection. The vials including the samples were sent to biomechanical laboratory for the ALP and ACP activity assay and stored at minus 20 degrees at which further processing could be carried out. ALP and ACP activity was assessed by colorimetric-DGKC and colorimetric-p-NPP method, respectively (Pars Azmon and Zist Shimi Co., Iran).

The data were statistically analyzed by means of repeated measures (ANOVA) and paired t-test using SPSS (Version 11). P value of <0.05 was considered as statistically significant. 

## Results

The clinical parameters of periodontal health of selected samples had similar score for all three groups at baseline (T1). The probe depth parameter also remained unchanged in all groups at T2 and T3. However, the plaque index and bleeding on probe index showed increased at DC and CC groups in both time intervals (* p *< 0.05). The statistical analysis revealed that the subjects might demonstrate significant differences in GCF ALP and ACP activity levels among the time points (T1, T2 and T3), and the treatments (distalization versus no orthodontic movement) and the sites (mesial versus distal).

**ALP activity**

**Different treatments**

At baseline, in both mesial and distal sites, ALP activity was similar among the 3 groups without significant differences (* p *= 0.65 and 0.89, respectively). At each later sampling time, in mesial and distal sites, statistically significant differences among the groups were seen. In mesial sites, statistically analysis showed an enzymatic activity significantly greater in the DC’s than in the CC’s and AC’s at both T2 and T3 (* p *< 0.001). Similarly, the ALP activity was significantly greater in distal sites of DC group compared with CC and AC groups at both T2 and T3 (* p *< 0.001). At mesial site, the difference between these measurements of AC and CC groups was not significantly different at both T2 and T3 (* p *= 0.072 and 0.062, respectively) (Figure 3). This data was similar for distal sites of AC and CC groups at both time intervals (* p *= 0.36 and 0.234, respectively).

**Different sites**

Result of the other statistical tests also showed a significantly greater enzymatic activity in distal than mesial sites of the DC group at both the 14^th^ day (T2), and the 28^th^ day (* p *< 0.01) (Figure 3). Conversely, in the CC group, no significant statistical difference in ALP activities was seen between the mesial and distal sites at different time points (T1, T2 and T3) (* p *= 0.48, 0.74 and 0.23, respectively) (Figure 3). Moreover, in the AC group, no significant statistical difference in ALP activities was seen between the mesial and distal sites at different time points (T1, T2 and T3) (* p *= 0.071, 0.072 and 0.13, respectively) (Figure 3). The peak level of ALP was observed in mesial sites (tension sites) of DC at day 14.

**Among time points**

The GCF ALP activity was increased significantly at mesial sites of DC groups between the time points (T1-T2) and (T2-T3) (* p *< 0.001 for both time intervals). However, at distal sites of DC groups, this increasing trend was only seen from T1- T2 (* p *< 0.001). The ALP activity decreased from T2-T3 which was not statistically significant (* p *= 0.163).

In contrast to T2-T3 time interval, the ALP activity changes at both mesial and distal sides of the CC groups were statistically significant between T1-T2 (* p *< 0.05). This enzyme activity at mesial and distal sites of the AC groups did not demonstrate a statistically significant change at any time interval (* p *= 0.145)

**ACP activity**

**Different treatments**

**Figure 4:**
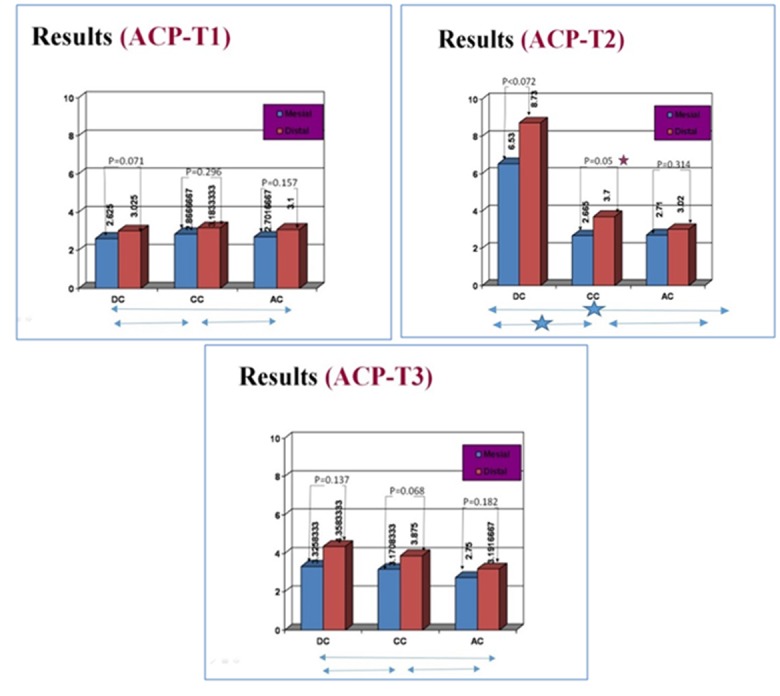
ACP activity (Mean) in GCF at mesial and distal sites of DC, CC and AC groups (n=12) at three time points (T1,T2 and T3).

Similar to the data for GCF ALP, the ACP activity at baseline was the same among experimental groups at both mesial and distal sites (* p *= 0.83 and 0.915). As it is shown at Figure 4, a statistically significant change in ACP activity was seen at T2 in both mesial and distal sites of DC group compared with the mesial and distal site of CCs and AC groups, respectively (* p *< 0.05). However, this difference was not observed between ACP activity levels of both mesial and distal sites of three groups at T3 (* p *= 0.596 and 0.257).

**Different sites**

Result of the other statistical tests also did not show a significantly greater enzymatic activity in distal than mesial sites of the DC and AC groups at both the 14^th^ day (T2), and the 28^th^ day (* p *= 0.072 and 0.137, respectively) (Figure 4). Conversely, in the CC groups, a significant statistical difference in ACP activities was seen between the mesial and distal sites at T2 (* p *=0.05). However, this significant difference was not observed at two other time points (T1, and T3) (* p *= 0.298 and 0.068, respectively) (Figure 4). The peak level of this enzyme was seen at the day 14. Moreover, in the AC group also, no significant statistical difference in ACP activities was seen between the mesial and distal sites at different time points (T1, T2 and T3) (* p *=0.157, 0.314 and 0.182, respectively) (Figure 4).

**Among time points**

The GCF ACP activity was increased significantly at both mesial and distal sites of DC groups between the time points (T1-T2) (* p *< 0.05) and decreased thereafter on both sites (T2-T3). However, the reported changes within two time intervals were not statistically significant at distal sites of DC groups (* p *= 0.257). At CC group, similar changing pattern was only seen at the distal sites that in contrast to mesial site changes. These changes were statistically significant at both time intervals (T1-T2 and T2-T3) (* p *< 0.05). The GCF ACP activity did not change within two time intervals at both sites of the AC groups (* p *= 0.867 and 0.243).

## Discussion

Our controlled longitudinal study has evaluated ALP and ACP GCF alterations at three different time points of orthodontic treatment. Although no differences were detected in the probe depth parameter between the groups, the plaque index and bleeding on probe index were greater in teeth receiving orthodontic appliance with or without force (CC and DC groups). Other studies have shown the similar result about gingival condition of teeth undergoing orthodontic treatment.(21-22) The underlying reason of observed differences could be the slight gingival enlargement following orthodontic treatment. Therefore, in studies evaluating the periodontal status of orthodontic patients, the site-specific characteristic of periodontal parameters and also the direct effect of reinforcement of oral hygiene instructions on periodontal condition of the teeth during orthodontic treatment should be considered.(23)

ALP has been reported as biologic marker of osteoblastic activity during bone deposition.(24-27) It is well proven that after inserting an orthodontic force, an early phase (3-5 days) of non-specific bone resorption activity is followed by an early (5-7 days) and a late phase (7-14 days) of bone deposition.(5) In the current study, high level of ALP activity was also seen on the 14 and 28 days (T2 and T3) in both mesial and distal of DCs which is supported by the approved hypothesis that described increased osteoblastic activity could take place in both tension and compression sites in alveolar bone.(5, 10, 14, 28) In contrast to controversial findings of animal studies evaluating ALP activity in periodontal ligament, the result of this study is in accordance with previous longitudinal studies on human subjects.(5, 29) Some previous studies observed that the peak of ALP activity occurred at day 14 whereas in our study the peak was seen at the day 14 and 28 at mesial and distal sites of canine retraction groups, respectively.(5, 9, 26) Some other investigations also reported bone formation appeared to begin after osteoclastic resorption phase that could last from 10 days to 3 weeks.(28, 30) These studies might therefore support the observed increase in GCF ALP in the DC group at T2 and T3. However, the observed difference can be attributed due to different force magnitude on teeth in different studies and also the limited duration of study.(10, 14) Greater ALP activity, though not statistically significant, in the CC group than AC group can be attributed to several subclinical displacements and movements that exerted by the wire in first step of leveling and alignments(5, 27) and also the dental plaque accumulation due to orthodontic appliance presence. (6, 12, 31)

The greater ALP activity values reported for the mesial (tension) sites compared with distal sites (compression) at DC group in both T2 and T3 might be as a consequence of greater bone deposition process over the resorption process in tension site.(30) The results obtained in the present study, which are in agreement with other studies, suggest that the increase in ALP activity in GCF might be related to dental site bone remodeling following orthodontic forces.(4, 14, 29-30)

ACP also has been reported as a biologic marker of bone destruction.(4) Data from our study, as well as other researches, have shown that in the early phase of tooth movement, the ACP activity is greater than later phase that follows by increasing in the ALP activity and decreasing in the ACP activity.(4-5) In this study, in contrast to Insoft *et al.* investigation,(4) the peak of ACP concentration was observed at day 14 (T2) that followed with a significant drop at the day 28 (T3); the maximum ACP activity was reported between 3 and 6 weeks in Insoft *et al.* study. These differences could be due to several different variables including small sample size of named study which restricts the ability to perform statistical analysis on the longitudinal data (3 cases), the applied force direction (buccolingually) and also lower force level (100g) compared to the present study (approximately 250g).(14) Moreover, in the present study, ACP activity was much greater in distal sites in DCs compared with the mesial sites of all groups at T2. However, this difference was statistically significant only at CC group at this time point. This observed higher level of ACP activity at distal sites of CC group, compared to mesial sites, can be attributed to higher level of subclinical periodontal plaque accumulation. This hypothesis is supported by investigations reported by Tynelvs-Brathal *et al.* who reported the positive correlations between ACP levels and inflammation in Beagle dogs.(32) However, since in our study the control of subclinical periodontal plaque accumulation was dependent on patients’ cooperation on received oral hygiene instructions, we cannot spectacle any data to support that presumption.

From a longitudinal point of view, present results showed a change in enzyme activity of ACP in the CC group during the study period, though this change was much greater in the DCs group. Our data showed a non-significant change of ACP activity at distal sites of DC and CC groups. The underlying reason for this observation might be that distal sites remained the main site of bone resorption in these groups.

This study showed that monitoring ALP and ACP activity at different sites of teeth undergoing orthodontic tooth forces could help the clinicians to better understand the local biological process. Longitudinal studies of orthodontic patients with history of gingival and periodontal conditions by means of phosphatase monitoring also can increase our knowledge about possible positive effect of orthodontic tooth movement on bone reconstruction of patients with controlled periodontal condition. 

## Conclusion

Alterations of ALP and ACP activities in human GCF during orthodontic tooth movement may reflect the metabolic changes in periodontium. Although, changes in ACP activity in GCF may be considered to be an indicator of tissue modification during orthodontic tooth movement in human, it appears not to be sensitive as ALP in distinguishing between tension and compression sites after the treatment. Since other factors such as different clinical conditions may influence the enzymatic activities, the ALP and ACP of human GCF should be considered as reliable biomarkers of tissue responses to orthodontic tooth movement, only when the oral hygiene is kept under control. 
